# The VACStent trial: combined treatment of esophageal leaks by covered stent and endoscopic vacuum therapy

**DOI:** 10.1007/s00464-023-09861-7

**Published:** 2023-01-13

**Authors:** J. Lange, G. Kähler, J. Bernhardt, J. Knievel, A. Dormann, U. Hügle, C. F. Eisenberger, M. M. Heiss

**Affiliations:** 1 Department of Abdominal, Tumor, Transplant and Vascular Surgery, Cologne-Merheim Medical Center, University Witten/Herdecke, Ostmerheimer Str. 200, 51109 Cologne, Germany; 2 Multispecialty Endoscopy Center, Mannheim Medical Center, University of Heidelberg, Mannheim, Germany; 3 Department of Surgery, Klinikum Suedstadt Rostock, Rostock, Germany; 4 Trials Center, Witten/Herdecke University, Witten, Germany; 5 Department of Gastroenterology, Cologne-Holweide and Merheim Medical Center, Cologne, Germany

## Abstract

**Background:**

Endoscopic treatment of esophageal leaks, mostly by covered stents or endoscopic vacuum therapy (EVT), has largely improved the clinical outcome in the last decade. However, both techniques suffer from significant limitations. Covered stents are hampered by a high rate of migration and missing functional drainage, whereas endoluminal EVT devices are limited by obstruction of the GI tract. The new design of the VACStent makes it a fully covered stent within a polyurethane sponge cylinder, allowing EVT while stent passage is still open. Initial clinical applications have demonstrated the fundamental concept of the VACStent.

**Method:**

A prospective multicenter open-label study was performed with the primary endpoint safe practicality, complete leak coverage, and effective suction-treatment of esophageal leaks. Secondary endpoints were prevention of septic conditions, successful leak healing, and complications, in particular stent-migration, local erosions and bleeding.

**Results:**

Fifteen patients with different, mostly postoperative anastomotic leaks were enrolled in three centers. A total of 41 VACStents were implanted. The mean number of VACStents per patient was 2.7, with a mean duration of VACStent treatment of 15 days. The primary endpoint was met in all VACStent applications (41/41 implants), resulting in a leak healing rate of 80% (12/15 patients). Septic episodes were prevented in 93% (14/15 patients) and there was no mortality. There were no severe device-related adverse events (SADE) nor significant local bleeding or erosion. Minor stent-dislocation and migration, respectively, was observed in 7%. Oral intake of liquids or food was documented in 87% (13/15 patients). One anastomotic stenosis was seen during follow-up.

**Conclusions:**

VACStent treatment is a safe and effective treatment in esophageal leaks which can be covered by the sponge cylinder. Its application was described as easy and resembling that of conventional GI stents, with an impressive clinical success rate comparable to EVT outcomes. The VACStent offers a new option for clinical treatment of critical situations in esophageal perforations and anastomotic sutureline failures.

Esophageal perforations present a significant clinical problem, which in the past resulted in high mortality and morbidity [1]. Only endoscopy, which has increasingly replaced surgical approaches, has significantly improved outcome. The principle behind the treatment is to close the leakage by suturing, clipping or stenting and to drain the wound secretions as effectively as possible [2, 3].

Especially the onset and use of covered stents led to a paradigm shift toward endoluminal endoscopic therapy [4]. However, clinical success was limited by the high rates of migration and dislocation in more than 50% of cases, as well as by the lack of functional drainage of the stents [4, 6].

The principle of endoscopic vacuum therapy (EVT) represented a milestone, which translated the good outcomes of negative pressure wound treatment (NPWT) in topical secondary wound healing to the inside of the body or intestine [5, 6]. The initial development of endocavitary EVT in the treatment of wound cavities of anastomotic suture line failures in the rectum, was later applied as endoluminal EVT to the esophagus and upper GI tract [7]. A suction catheter attached to the sponge and the external pump applies a vacuum of 80–125 mmHg to the open-cell polyurethane sponge (PU) occupying the esophageal lumen at the leak. This suction produces a corresponding vacuum at the mucosa-sponge interface, which not only facilitates wound closure but also effective drainage of the inflammatory wound secretions [5, 7]. Furthermore, suction can condition the wound bed, improve perfusion, and effectively induce granulation tissue [7, 8, 11]. One drawback is that suction closes off the GI tract with the same force, so that passage is no longer possible. This prevents early enteral nutrition and limits the principle of endoluminal EVT to the upper GI tract.

The VACStent has now been developed as a new treatment option avoiding these drawbacks of endoluminal EVT by combining the latter with the benefits of covered stenting. The VACStent comprises a covered self-expanding nitinol stent (SEMS) encased in a polyurethane sponge cylinder. Only the flanged ends of the covered stent contact the intestinal wall, sealing it from the lumen when suction is applied.

Initial clinical applications have demonstrated that the VACStent does realize these design-related features clinically [9, 10]. The aim of this prospective multicenter trial was to evaluate how different endoscopists assess the clinical handling, whether the technical application is successful, and if this allows effective closure of various types of esophageal leaks. In addition, possible complications and the clinical course were to be studied as well.

## Method and materials

### Investigational device

**Fig. 1:**
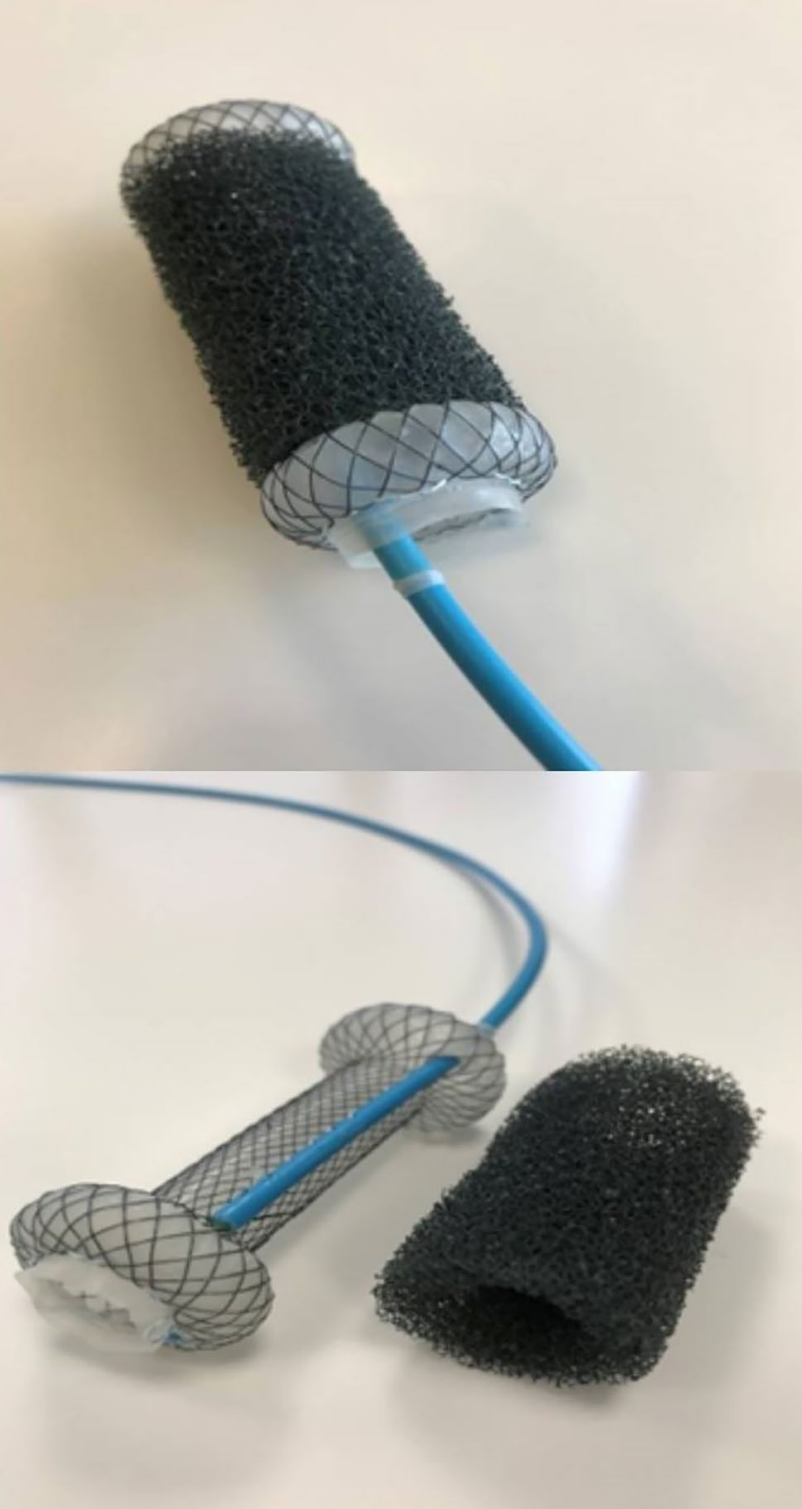
VACStent system consisting of a silicone-covered stent within a PU-sponge cylinder with an embedded suction catheter

The VACStent combines a fully covered intestinal stent surrounded by a polyurethane sponge cylinder (Fig. 1). A suction catheter (diameter 10F, length 1000 mm) within the sponge attaches to an adjustable vacuum pump, creating a negative pressure at the mucosal-sponge interface. This vacuum closes a perforation or leak off against the intestinal lumen and induces the drainage of inflammatory infectious secretions. Like a suction-cup it also immobilizes the covered stent very efficiently against the bowel wall. This should stop migration and slippage, the major limitations of fully covered stents when used for sealing intestinal leaks. Another major benefit of the VACStent is its ability to allow passage of nutrition and liquid under ongoing endoluminal EVT [10].

The VACStent is loaded into an introducer-system which can be applied endoscopically over-the-wire similar to other conventional SEMS. This introducer-system (diameter 4.2F, length 100 cm) comprises the loaded VACStent mounted on an inner catheter and constrained by an outer tube. By retracting the outer tube, the VACStent is released and expands to a dumbbell shape with an inner diameter of 14 mm. The flanged ends of the VACStent have a 30 mm lumen which frames the sponge cylinder against the intestinal lumen. This allows circular EVT over the full length of the sponge cylinder (50 mm). The VACStent is manufactured by VACStent GmbH (Fulda, Germany), distributed by Microtech-Europe GmbH (Düsseldorf, Germany) and has a European conformity certification (CE).

### VACStent application

**Fig. 2:**
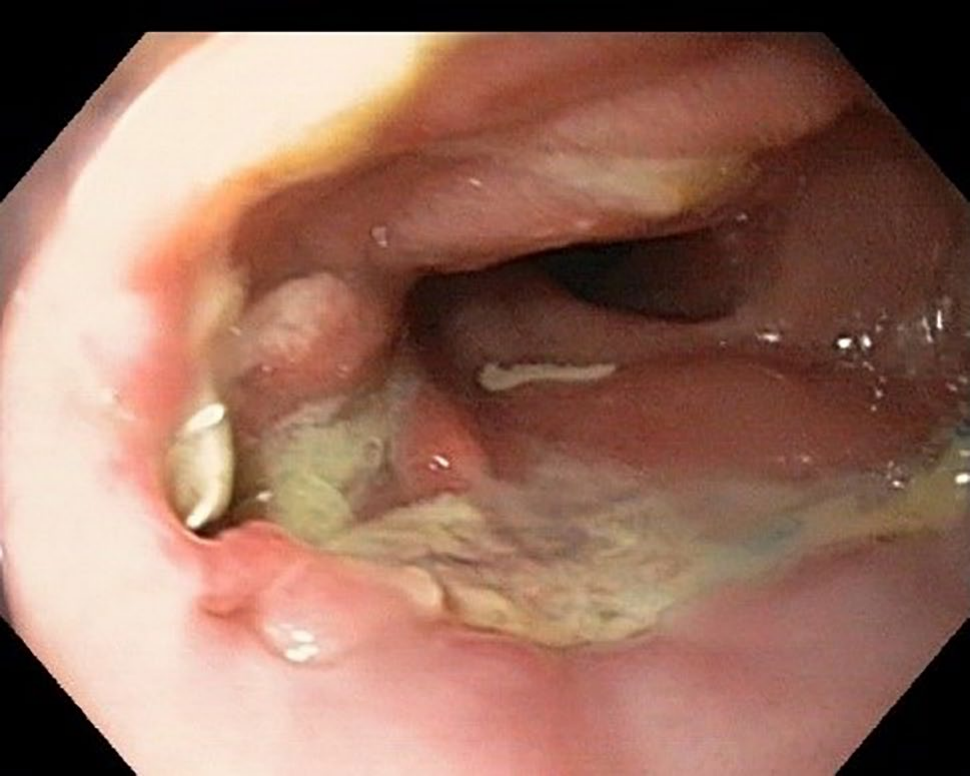
Insufficiency of a circular staple suture after esophagectomy with a transmural fistula at 8 o’clock

**Fig. 3:**
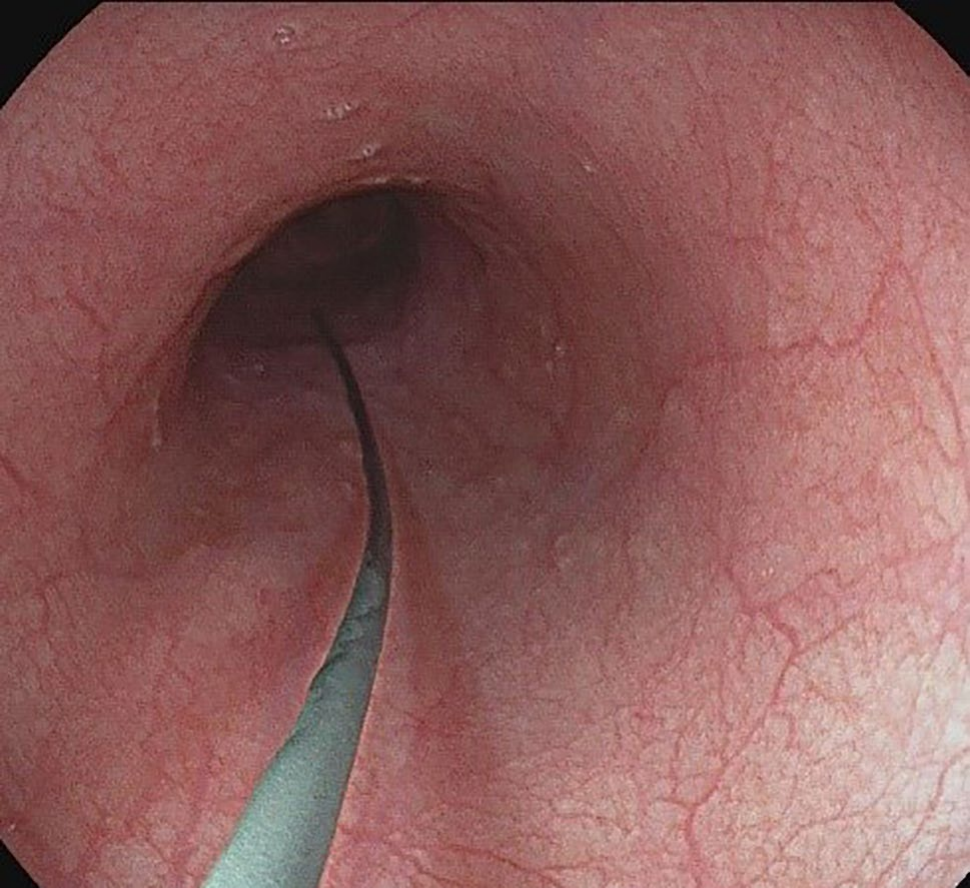
Iserted stiff guide wire distal to the anastomosis

**Fig. 4:**
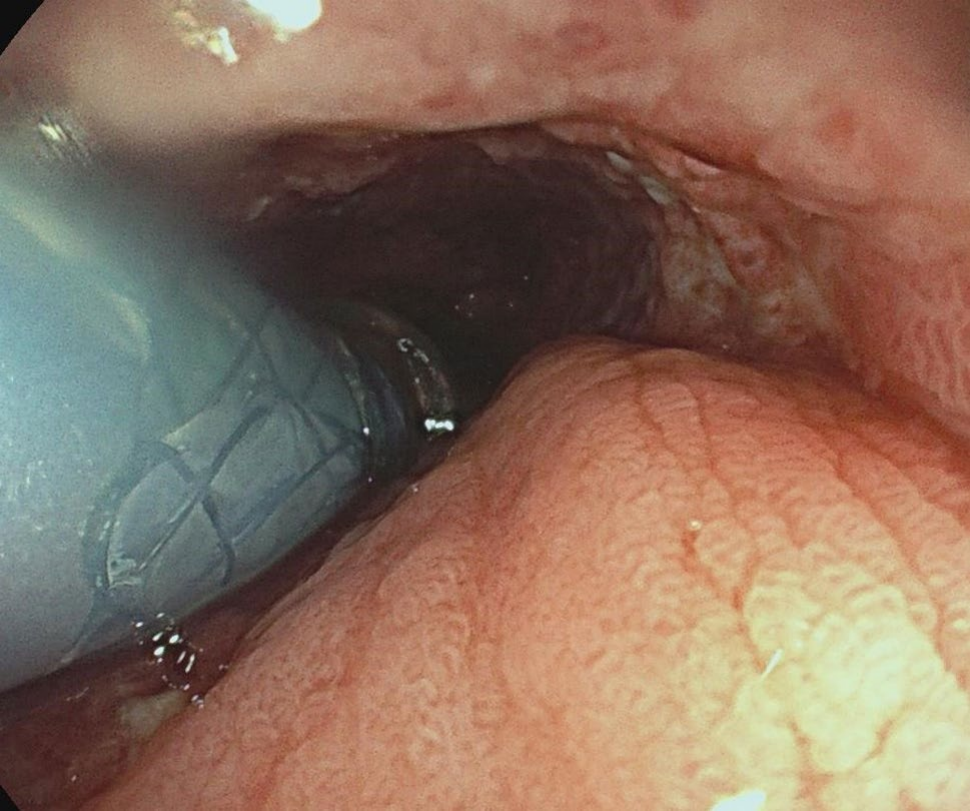
Endoscopic observation of the exact placement and release of the VACStent from the delivery system

**Fig. 5:**
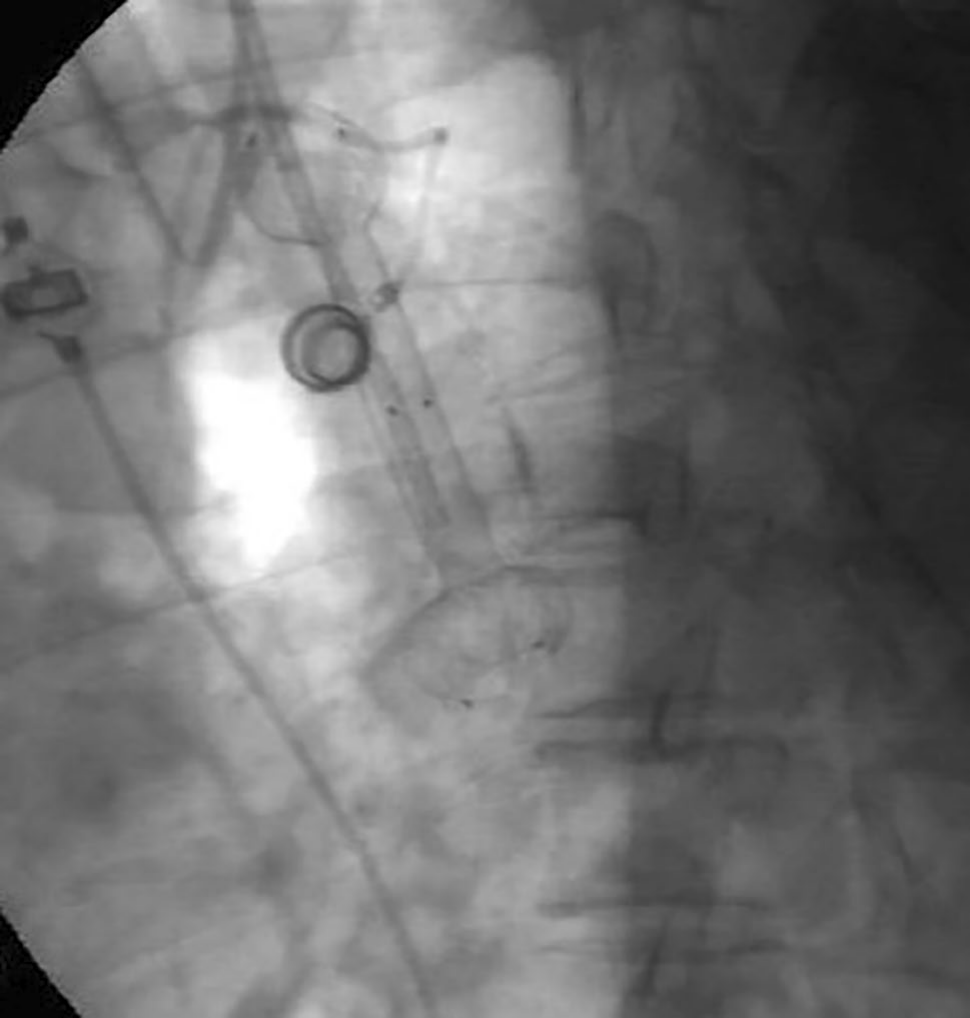
Alternative possibility of placement and release control oft he VACStent by fluoroscopy

**Fig. 6:**
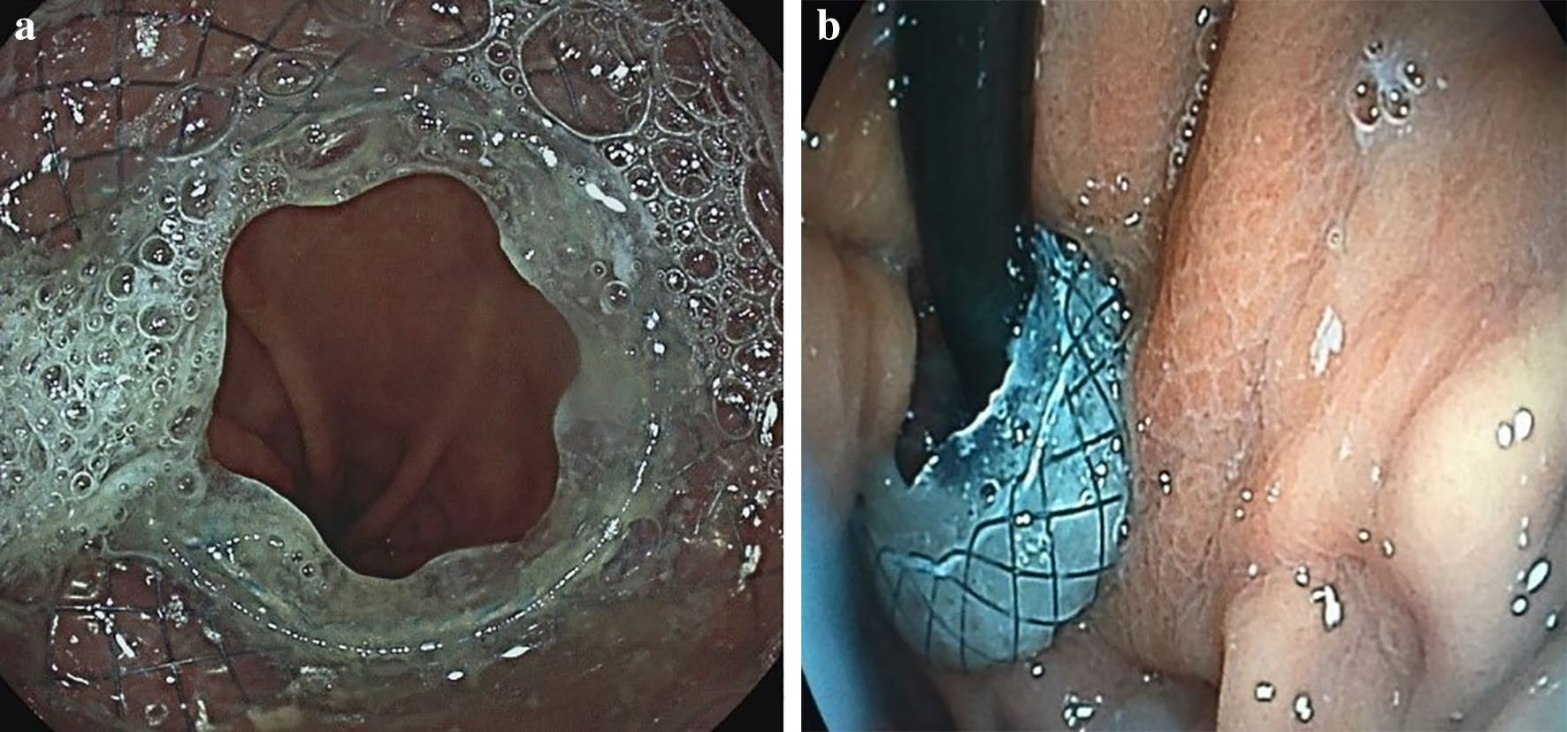
Free passage through the VACStent is checked endoscopically (**a**) and the exact positioning oft he distal VACStent can additionally be viewed in inversion (**b**)

Transoral endoscopy is performed to define the precise location and dimension of the leak (Fig. 2).The patient was eligible for VACStent treatment if the sponge (50 mm) was able to cover the leak completely. As a first step, a stiff guide wire was placed under direct vision in the stomach or bowel (Fig. 3). Then the delivery system was carefully advanced over-the-wire and the VACStent deployment observed via a small 8 mm endoscope, which paralleled the delivery system (Fig. 4). In some cases (9 patients), deployment was monitored by fluoroscopy to ensure the positioning and release of the VACStent (Fig. 5). Free passage through the stent can be checked endoscopically, as well as the exact positioning of the distal stent end (Fig. 6). The application system and guidewire were then removed and the suction catheter passed retrograde through the nose. Before connecting the suction catheter to a VAC-pump (e.g., Curasul®, BSN medical GmbH, Hamburg, Germany) with a plastic Y-adapter, retrograde rinsing with 20 ml 0.9% NaCl solution of the sponge cylinder was performed to facilitate and ensure the deployment of the open-cell PUR-sponge. This Y-adapter allowed easy connection to most of the clinically available vacuum pumps of various manufacturers.

A rating scale was designed to assess the endoscopist's subjective impression of the handling of the system. Since this differs little from that of a covered SMES, few problems occurred during handling and was mostly rated easy or moderate to handle (Table 2).

The recommended length of stay for a VACStent was 2 to 5 days, with an expected mean number of 3 stents necessary for successful healing of a leak. Before removal of the VACStent extensive retrograde rinsing of the sponge via the drainage-tube (at least 40 ml 0.9% NaCl) is recommended. Moreover, the suction should be stopped for at least 2 to 4 h before VACStent removal. Removal is performed endoscopically with forceps to pull at the retrieval loops placed at the ends of the VACStent.

### Study design

The study was a multicenter prospective single arm, open-label feasibility and safety study to treat leaks in the upper GI tract with the VACStent combining the principle of EVT and covered nitinol SEMS. The VACStent treatment was performed by experienced endoscopists at three German tertiary centers (Klinikum Rostock-Süd, University clinic Mannheim, Klinikum Köln). The ethics of this trial adhered to the World Medical Association Declaration of Helsinki and was approved by the Institutional Review Board (IRB) of Witten/Herdecke University (No. 124/2018) and other appropriate IRBs. The trial was registered with the DRKS (German Registry of Clinical Trials) under the identifier DRKS00016048.

### Trial endpoints

The primary aim of the trial was to demonstrate the safe technical practicality of the VACStent and its successful coverage of leaks. The endpoint was achieved in full if the VACStent could be deployed, positioned and continuous suction via the sponge cylinder applied. This was assessed by endoscopy during the procedure and continuous control of the established negative pressure. The secondary objectives were to assess any septic signs during the clinical course, morphological healing of the leak, and complications.

### Patient collective

Eligible was any patient with endoscopic conformation of a diagnosis of upper GI leak, either postoperatively at an anastomosis or an iatrogenic leak caused by an endoscopic (e.g., TEE) or surgical procedure, and that this lesion was reachable by the applicator-system of the VACStent, provided that informed consent had been given.

Excluded were patients with leaks not within the endoscopic accessibility of the VACStent, clinically unstable septic patients requiring urgent surgery to treat the septic focus immediately, patients with a full stomach and/or severe permanent vomiting with clinical ileus signs, and patients needing full anticoagulation or with thrombocytopenia < 20.000/µl.

### Data collection and analysis

Safety, efficacy, and clinical course of the VACStent treatment were analyzed daily from patient enrollment until hospital discharge. during follow-up visits study protocol recommended control endoscopy at 30 days after discharge, after 6 months and after 12 months. In 13 of 15 patients at least one follow-up endoscopy for long-term data of the VACStent treatment was performed. All data were collected in a case report form (CRF), entered in a data base, and analyzed with the SPSS statistical software package [14].

### Outcomes

#### Patient characteristics and performed procedures

In total, 15 patients in three sites in Germany (5 in Cologne, 3 in Rostock, 7 in Mannheim) had been enrolled in the trial between August 2019 and November 2020 (Table 1).

**Table 1:** Patient population

Pat. No	Age, sex	ASA	Disease/procedure	Localisation of leakage	Leakage size BxHxT, cm	Days after surgery	Endoscopic treatment before stenting	VACStent, numbers	Duration of VACStent treatment, days	Suction/pressure (median), mmHG	Complication	Clinical success	Oral intake during VACStent
1	75, m	3	Lung resection for bronchial Cancer	Esophagus at 29 cm from teeth row, right side wall	1.5 × 1× 1	17	Endoluminal –Esosponge	2	10	100	None	Yes	Yes, liquids & solid food
2	75, w	2	LINX band explantation for dysphagia	Distal esophagus supracardial	1 × 0.5× 1	n/a	None	1	4	80	None	Yes	Yes, liquids
3	64, m	2	Distal thoracoabdominal esophagectomy with gastric conduit for esophageal cancer	Esophago-gastric anastomosis	0.5 × 0.5 × 1	12	None	3	9	80	Aspiration during VACStent extraction	Yes	Yes, liquids
4	59, m	1	Pneumatic balloon dilatation of distal esophageal sphincter for achalasia	Distal esophagus	2 × 3 × 2.5	n/a	None	2	8	75	None	Yes	Yes, liquids & solid food
5	50, m	3	Laparoscopic sleeve gastrectomy for morbid adipositas	Distal esophagus/gastric junction (combined fistula cardial/subcardial "His fistula")	2 × 3 × 2.5	34	Endoluminal esosponge	7	43	75	Stent migration	No	Yes, liquids & solid food
6	60, m	3	Thoracoabdominal esophagectomy with gastric conduit for esophageal cancer (Ivor-Lewis)	Upper esophagus at 20 cm from teeth row at the esophageal-gastric anastomosis	0.4 × 0.4 × 1	23	None	9	55	75	Persisting esophageal-tracheal fistula; stent migration	No	Yes, liquids & solid food
7	67, m	1	Thoraco abdominal esophagectomy for Barrett's carcinoma	Esophageal anastomosis at 26 cm from teeth row	0.5 × 0.3 × 0.5	10	None	2	10	75	None	Yes	Yes, liquids & solid food
8	62, w	2	Thoraco abdominal esophagectomy pleurectomy, for esophageal cancer	Directly at the circular stapler anastomosis at 25 cm from teeth row	3 × 1 × 2	33	None	1	6	80	None	Yes	Yes, liquids
9	76, m	3	Abdomino-thoracic esophageal partial resection with tubular gastric elevation for AEG 2 cancer	Chronic fistula at the esophageal-gastric anastomosis	0.2 × 0.2 × 8	150	Endoluminal esosponge	3	13	100	None	Yes	Yes, liquids
10	74, m	2	DaVinci-assisted abdomino-thoracic esophagectomy with gastric conduit for esophageal cancer	At the esophageal-gastric anastomosis at 26 cm from teeth row	1.5 × 1.5 × 6	20	Intracavitär esosponge	1	7	80	Stenosis 30 days after discharge	Yes	Yes, liquids & solid food
11	91, m	3	Latrogenic esophageal perforation during ERCP manouver	Esophageal laceration at 25 cm frum teeth row	3 × 3 × 6	28	Esophageal stenting, endoluminal esosponge	1	5	80	None	Yes	Yes, only liquids
12	84, w	3	Transhiatal distal esophageal resection with gastric conduit for cancer of the gastroesophageal junction	Front wall slightly left at the anastomsis	2 × 0.8	2	Endoluminal esosponge	2	11	100	Pneumonia	Yes	No, only via gastric tube
13	65, m	3	DaVinci-assisted abdomino-thoracic esophageal resection with gastric elevation	Insufficiency of esophagogastrostomy at 30 cm from teth row	1.5 × 1.5 × 5	4	Intracavitary esosponge	2	11	100	None	Yes	Yes, liquids & solid food
14	83, w	2	Esophageal perforation during TEE	20 cm fromteeth row, 2 cm aboral of the superior esophageal sphincter	1 × 1 × 7	n/a	None	3	19	125	None	Yes	Yes, liquids & solid food
15	73, w	3	Lap. Repositioning of enterothorax anterior and posterior hiatoplasty, 360 °C fundoplication	Large cavity with secondary pleural emyema	2.5 × 1 × 10	10	None	2	9	100	Termination of endoscopic therapy	No	No, only via gastric tube

9 patients were diagnosed with malignancy, 8 of them underwent resection in esophageal cancer, one was resected in lung cancer with invasion of the esophagus. In one patient with resected esophageal cancer a small esophageal-tracheal fistula developed at a very high intrathoracic anastomosis (20 cm from the incisors). VACStent treatment was started on postoperative day 23. The patient was clinically stable and could swallow liquid and mashed food during the treatment with consecutive 9 VACStents over 55 days. However, the fistula did not heal completely and was finally closed by surgery.

One patient had a leak after repositioning of an up-side-down stomach together with parts of colon and small intestine and performing hiatoplasty together with fundoplication. After 9 days of uneventful VACStent treatment of the luminal leak a large thoracic abscess behind it was diagnosed and treatment was changed to esophageal resection and empyema evacuation.

In one patient, a gastric sleeve conversion to a gastric bypass developed subsequent multiple anastomotic fistulas. This patient was treated first by laparoscopic revision and intraluminal EVT with a sponge (Eso-Sponge®, BBraun. Melsungen, Germany). Despite 34 days of Eso-Sponge® treatment the fistulas persisted and were then treated by the VACStent. This was successfully continued for 43 days, allowing oral nutrition. However one clinically inapparent fistula was left untreated and finally closed spontaneously several weeks later.

Three patients had suffered iatrogenic perforation, one during ERCP, one during pneumatic dilation in achalasia, and one during transesophageal endoscopic echocardiography (TEE).

In one patient an invading LINX band was removed surgically, and the remaining transmural gap closed by intraoperative application of the VACStent.

#### VACStent treatment

In 15 patients diagnoses with a leak in total 41 VACStents were placed endoscopically, by 5 experienced endoscopists, all with a personal expertise of more than 1000 endoscopies per year and experience in using covered SEMS and PU-sponge EVT. Placement was reported to be easy or only moderately difficult (Table 2). In all cases correct positioning and deployment of the VACStent was technically successful. The leak was covered completely, and in all cases continuous suction with a mean suction of − 85 mmHg (range − 75 to − 125 mmHg) was installed. The primary endpoint was met in all patients (Table 3).

**Table 2:** Results: VACStent applicability

	N (%)
Patients treated (n)	15 (100)
VACStent placements	41 (100)
Median implantation time (min)	32 (10–65)
Median no. VACStents/patient	2 (1–9)
Median total hospital stay (days)	10 (4–55)
Ease of placement	
Easy	33 (80)
Moderate	8 (20)
Difficult	0 (0)
Impossible	0 (0)
Leak coverage	
Easy	32 (78)
Moderate	7 (17)
Difficult	2 (5)
Impossible	0 (0)
Vacuum application: median negative pressure in mmHg	80 (75–125)
Easy	29 (71)
Moderate	11 (27)
Difficult	1 (2)
Impossible	0 (0)
VACStent removal	
Easy	34 (83)
Moderate	4 (10)
Difficult	3 (7)
Impossible	0 (0)

**Table 3:** Results: study endpoints

Items	N (%)	
Primary endpoint		
Technical success/leak coverage/suction application	41/41 (100)	
Secondary endpoints		
Clinical signs of sepsis	1/15 (7)	
Mortality	0/15 (0)	
Complete closure of leak	12/15 (80)	
Oral intake of liquids	13/15 (87)	
Oral intake of solid food	8/15 (53)	

In all cases, the septic focus of the leak was controlled by the VACStent treatment. In one patient the clinically septic condition worsened later on during the trial due to secondary pleural empyema. There was no death recorded until hospital discharge of the patients.

Complete morphological healing of the leak was seen in 12 out of 15 patients (80%). As noted above, in 2 patients the persistent leak was closed by surgery, while in one patient the fistula closed spontaneously after the VACStent treatment.

The mean VACStent treatment time was 15 days (range 4–55), the mean indwelling time for the VACStent was 5 days (range 2–8). The mean number of VACStent applications per patient was 2.7 (range 1–9). VACStent implantation took an average of 34 min (SD14), and VACStent removal took 28 min (SD 8). VACStent replacement took an average of 38 min (SD17), which was only 10 min longer. These times reflect the difficulty of the procedure and do not suggest a relevant demonstrable learning curve.

In 13 out of 15 patients (87%) oral intake of water and liquids was possible. In two patints a gastric tube was inserted through the VACStent distally and enteral tube feding was performed.. One patient showed impaired swallowing probably caused by a neurological condition, the other patient was intubated and ventilator-supported. Eight patients reported no problems with the gradual return to more solid mashed food. The deployed VACStent could also be passed with a small endoscope (8 mm) allowing investigations and manipulations distal to the VACStent (Fig. 6).

In no case was a severe adverse device associated event (SADE) reported (Table 4). In particular, a VACStent migration or dislocation was observed only in 7% throughout the trial. Moreover, no clinically significant severe erosion or ulcer was noted and also no local bleeding, neither throughout the VACStent site nor in the wound cavity. Also, no significant malfunctions of the drainage capacity of the VACStent were reported.

**Table 4:** Results: VACStent complications

	N (%)
Dislocation/migration	
None	38 (93)
Rarely	3 (7)
Significant	0 (0)
Massive	0 (0)
Erosion/ulcer	
None	32 (78)
Rarely	9 (22)
Significant	0 (0)
Massive	0 (0)
Local bleeding	
No	36 (88)
Rarely	5 (12)
Significant	0 (0)
Massive	0 (0)

Removal of the 41 VACStents was without major problems, but tissue ingrowth was reported in three patients treated with higher suction and/or longer VACStent indwelling time. In all cases intense retrograde rinsing of the sponge cylinder through the suction catheter was able to loosen the sponge fixation. In addition, the suction pump was stopped at least two hours before VACStent removal. In one of the three patients the sponge cylinder was lost during extraction, but could still be removed without problems.

In order to prevent any aspiration during the endoscopic procedure, the type of anesthesia was left to the discretion of the local endoscopist and anesthetist. and 27% of the patients underwent VACStent treatment under general anesthesia with intubation. However, as the easy application procedure compared quite well with conventional SEMS, analgo-sedation with propofol was performed in 73% of stable and conscious patients. Minor aspiration during VACStent procedures, treated by endobronchial lavage and suction, was seen in just two cases out of 41 procedures. No patient experienced post-procedural clinical pneumonia.

Follow-up was done endoscopically in 13 patients and revealed one anastomotic stenosis with dysphagia complaints 30 days after patient discharge. In all other patients no clinically significant dysphagia was observed.

## Discussion

In this prospective multicenter feasibility study, the VACStent proved that it can combine successful endoscopic vacuum therapy of upper GI tract leaks and anastomotic suture line failures, with the benefits of stent therapy. In all 41 cases, the VACStent treatment was performed without any significant problem. After release and deployment of the VACStent, the leakage was successfully covered and the drainage function of the PU-sponge cylinder activated after connection to a vacuum pump. Therefore, the primary endpoint was achieved in all 41 VACStent applications.

With regard to the most essential clinical success parameter, the prevention or improvement of septic patient constellations, the VACStent application was mostly successful in all 15 patients. In one patient the symptoms worsened later on due to secondary pleural empyema requiring surgical treatment.

Definitive morphologic healing was observed in 80% of patients after an average of 15 days of VACStent placement. This corresponds to a healing rate as can be expected with EVT through a PU sponge [6, 13]. However, the healing rate is affected by the chronicity of an existing leak or fistula, the site of the defect, and the applied suction vacuum [13]. Previous clinical experience with PU-sponge therapy is reflected in the outcomes of this trial. Two patients with long-standing chronic fistulas for 34 and 23 days, respectively, were successfully covered clinically by the VACStent, but did not heal completely.

Deeper and more complex wound cavities can be treated with the VAC-stent if the connection (e.g.fistula) to the leakage is sufficiently large so that it does not collapse due to the applied suction. Then the negative pressure prevails in the wound cavity, mobilizes and drains the wound secretion. In the case of very large wound cavities, pretreatment or synchronous application of an intracavitary PU-sponge can also be performed. The advantage of the additional VAC-stent application is the direct wound closure toward the intestinal lumen without impairing the drainage function or without suctioning intestinal contents/nutritional components into the sponge.

Besides the effect of fistula duration and fistula constellation, the suction level of the vacuum pump is probably important [13]. Thus, EVT has been shown to be able to heal a leak even at low vacuum of − 60 mmHg but this was associated with a lower induction of granulation tissue. On the other hand, suction level also correlates with the phenomenon of granulation tissue ingrowth into the open-cell PU-sponge, especially when the indwelling time of the VACStent exceeded 4 days [15, 16]. To loosen the sponge cylinder, it has proven effective to terminate suction at least two hours before VACStent removal. In addition, intensive retrograde irrigation of the sponge cylinder with 0.9% saline and careful endoscopic loosening the sponge cylinder along its entire length. Overall, removal of the VACStent was judged by the investigators to be easy in 83% of cases (Table 2).

A similar assessment was made by the investigators for implantation of the VACStent, with insertion and release judged to be easy in 80% and moderately difficult in 20%. In two cases, coverage of the leak was perceived as difficult, but was ultimately performed successfully. Control of VACStent delivery was performed endoscopically, with the endoscope inserted transorally into the esophagus in addition to the recumbent delivery system. Compared with VACStent delivery under fluoroscopy, this has the benefit of direct vision of correct VACStent release. In addition, the VACStent can thus be applied and exchanged without the need for fluoroscopy, e.g., on an intensive care unit.

Overall, manageability and application of the VACStent system was very easy and did not differ significantly from conventional stent systems. This implies a low learning curve and thus safe application of the VACStent System. Removal during stent changes was also usually without problems, if the endoscopist was experienced in handling the EVT with endoscopically placed PU-sponges. This was especially true for the observed complications, which usually were rare and then proved to be easily correctable.

Severe VACStent-associated complications (SADE reports) did not occur in any of the 41 VACStent treatments. Likewise, continuous suction via the PU-sponge cylinder was found unproblematic throughout the treatment period. Only in one case was this considered difficult. This continuous suction on the esophageal/intestinal wall leads to a "suction cup" effect that reliably prevents the main problem of covered stents‒migration and dislocation. Minor dislocation during VACStent implantation occurred in only three cases (7%). In 22% (9/41) of the cases, local moderate erosions or ulcers were observed in the area of the stent beads without resulting in perforation or bleeding. Local low-grade bleeding from the granulation tissue was rare and only seen in 12% (5/41) of cases and remained without clinical significance or need for intervention.

A major benefit of the VACStent design principle is the free passage through the VACStent body; thus, in 87% (13/15) early oral liquid and food uptake was possible. In 8 patients, mashed food was also started, which was possible without any aspiration or stent dislodgement. Due to the lack of peristalsis in the area of the VACStent, the passage is, of course, restricted, but still possible for liquids and strained food similar to what is seen with conventional covered stents.

Due to the special design of the VACStent, there was no firm attachment between the internal silicone membrane and the external nitinol wires in the cylindric part of the stent. Depending on the position and curvature of the stent, this results in longitudinal folds protruding into the lumen and thus visually constricting the cross-section. However, as stated above, this did not lead to any noticeable significant functional limitation.In another recently published trial with the VACStent for esophageal leaks, however, this phenomenon was described by the authors as problematic and preventing oral nutrition [17]. Our clinical observations did not confirm this. Nevertheless, this problem was taken up by the manufacturer and has been corrected, and the current model of the VACStent no longer exhibits luminal narrowing due to longitudinal fold formation in the covering.

With regard to the duration of the VACStent treatment and the observed healing rate, further significant differences between the two trials became apparent. In Chon's trial,. the feasibility was tested in 80% of all patients by applying just one VAC-Stent. Only in 4 patients also a second VACStent was used but no one more. In our study the used approach was to treat as long as necessary to close the leak also morphologically. This resulted in a threefold number of applied VACStents (2,7 vs 1,2), a threefold longer VAC-Stent laytime of 14,2 vs 4,8 days and an increased leak closing rate of 80% vs. 60%. Another difference is the low suction pressure of 65 mm Hg versus 85 mm Hg in our study, because suction strength correlates with granulation tissue induction and healing speed.However, due to the functional resemblance of the VACStent with the intraluminal EVT using a PU-sponge, a significantly longer treatment period of 14–28 days, on average, would be expected until possible healing of the leakage could be observed [13, 15, 16]. The results show, that even with such a short treatment period, the EVT effect of the VACStent resulted in improved and accelerated leakage healing. Thus, these clinical results are comparable to ours, considering that our trial saw a healing rate of 80%, with a mean of 2.7 VACStents per patient, and a treatment duration of 14.2 days. The easy handling and low complication rates of the VACStent were similar in both studies. Furthermore, a major difference is the fact that this feasibility study was performed not monocentric but multicentric in 3 centers by a total of 5experienced endoscopists, and thus may better reflect the criteria of VAC-Stent handling.

Esophageal stenosis with clinical dysphagia must be discussed as a long-term sequela of endoluminal EVT, and thus possibly VACStent therapy [6]. Cicatricial stricture developed in one case, which was resolved without problems by dilatation. This is consistent with findings reported for EVT and PU-sponge treatment, that there is no procedure-associated increase in the rate of stenosis [6]. Stenosis is mainly determined by the extent of the necrosis or wound cavity and the wound dehiscence [11].

The disadvantages of the VACStent compared to the conventional covered SEMS are the need for a stent change after 4–8 days and the transnasal suction catheter with a vacuum pump. The VAC-stent is not suitable as sole therapy in complex wound cavities without wide access to the esophagus. The VAC-stent is also not suitable for very high cervical anastomoses. Costs are higher than conventional SEMS, as an average of 2.7 stents were required per treatment. Compared to sponge EVT, costs are also higher, but due to the VACStent-associated wound closure, changes are needed much less frequently.The trial outcomes demonstrate that the VACStent can be used for leaks that can be covered with the sponge cylinder. Besides iatrogenic injuries (e.g., during ERCP, TEE), these are mainly anastomotic suture line failures after esophageal resections or in bariatric surgery. However, due to the limited study size the efficacy of this new approach has to be further evaluated especially in comparison to other treatments like conventional EVT, stents, clips or sutures. It has been shown that the VACStent should be employed as early as possible, preferably at the time of diagnosis, to prevent the formation of larger wound cavities and chronic fistulas. Especially the immediate application in fresh endoscopic or surgical lesions leads to healing within a few days [10]. This observation, together with the wound healing-promoting effect of EVT, led to the concept of prophylactic intraoperative EVT after esophagectomy [18]which can also be achieved with the use of the VACStent.

Due to the fact that the trial was designed as a multicenter feasibility study there are several limitations including the large heterogeneity of patients and treatment parameter, and the lack of comparison with other therapies.

In conclusion, the VACStent has proved to be a new medical device capable of combining the benefits of EVT with those of stenting while being simple and safe to apply. The VACStent allows immediate wound closure and effective drainage of endoluminal wound cavities and is therefore able to control the septic focus and promote and accelerate morphologic healing. The open passage through the VACStent allows for rapid postoperative nutrition and endoscopic access distal to the leak. The design and mechanism of action, along with the ease of use, show promise that the VACStent might have potential to improve clinical outcomes in resections and perforations of the esophagus and bariatric surgery.
